# SARS-CoV-2 and *Plasmodium falciparum* Co-Infection in a Returning Traveler

**DOI:** 10.3389/fpubh.2022.871374

**Published:** 2022-08-05

**Authors:** Qian Huang, Wen-Jie Xu, Xiao-Xiao Wang, Xuan Zhang, Ke-Nu Pan, Jia-Qi Zhang, Hua-Liang Chen, Wei Ruan, Li-Nong Yao

**Affiliations:** ^1^Department of Infectious Diseases, Hangzhou Xixi Hospital, Hangzhou, China; ^2^Department of Infectious Diseases, Zhejiang Provincial Center for Disease Control and Prevention, Hangzhou, China; ^3^Medical Laboratory, Xixi Hospital of Hangzhou, Hangzhou, China

**Keywords:** COVID-19, Sub-Saharan Africa, co-infection, *Plasmodium falciparum*, malaria, SARS-CoV-2

## Abstract

Since December 2019, the Coronavirus Disease 2019 (COVID-19) pandemic has become a non-neglectable context for the whole healthcare system. Under the background of COVID-19, the detection and diagnosis of malaria cases are under challenge. Here, we reported a COVID-19 and malaria co-infection traveler who has a long living history in Cameroon. The case was administered with dihydroartemisinin and piperaquine tablets for malaria, Lopinavir and Ritonavir tablets, Arbidol, recombinant human interferon α-2b and Compound Maxing Yifei mixture for COVID-19, and Zolpidem Tartrate tablets, Diazepam, Paroxetine Hydrochloride tablets, Thymosin α1, and Lianhua Qinwen Jiaonang during the second hospitalization of the patient since the patient has a certain level of anxiety and insomnia with no evidence of inflammatory reactions. After being tested negative two times for severe acute respiratory syndrome coronavirus 2 (SARS-CoV-2) in 48 h, the patient met China's COVID-19 discharge standards and was discharged with stable vital signs and mental state. Since most countries in the sub-Saharan region have a fragile health system, co-infection for both *Plasmodium* and SARS-CoV-2 may not be uncommon, and raise a challenge in diagnosis, treatment, and prevention for both diseases. We add to the literature on co-infection of *P. falciparum* malaria and COVID-19 and offer operational advice on diagnosis, prevention, and treatment for the co-infection.

## Introduction

A novel severe acute respiratory syndrome coronavirus-2 (SARS-CoV-2) was emerged in December 2019, causing a new infectious disease named Coronavirus Disease 2019 (COVID-19). Till May 2022, more than 512 million COVID-19 cases have been confirmed worldwide (1). Malaria has always been a major public health problem, affected 241 million people in 2020 and caused 627,000 deaths. The COVID-19 pandemic is a great challenge for the diagnosis and treatment of malaria, especially in areas with limited medical resources. According to the estimation from World Health Organization (WHO), about two-thirds of increasing deaths (47,000/69,000) were due to disruptions during the COVID-19 pandemic. Africa has always been a hotspot for malaria (2), some of them relying much on international funding whose disease program might be stuck because of the fund interruption (3). These countries were facing challenges in the diagnosis and treatment of both diseases. The possibility of co-infection of COVID-19 and other pathogens might increase the difficulties of this procedure (4). Considering the high disease burden of malaria in Africa and south-eastern Asia, malaria and COVID-19 co-infection could be a public health problem, not only because of the potential numerous patients but also because of the raising difficulties of diagnosis, treatment, and management of the patients. Following a 70-year effort, China has not reported indigenous malaria cases since 2017; on 30 June 2021, the WHO announced that China had been certified malaria-free. The focus of malaria control in China has shifted to the prevention of local re-transmission of imported malaria. Here, we report a patient co-infected with SARS-CoV-2 and *Plasmodium falciparum*. The patient had a long history of living in Cameroon and was diagnosed in China. This case adds to the literature on *P. falciparum* malaria and SARS-CoV-2 co-infections.

## Materials and Methods

### Case Detection

Malaria case was defined based on a unified national criterion, according to clinical symptoms, and traveling history confirmed and classified by laboratory methods. If the patients with malaria-like symptoms and a history of visiting malaria-endemic areas were discovered by a medical institution in China, they will undergo laboratory tests, such as Rapid Diagnostic Test (RDT), microscopy, and polymerase chain reaction (PCR) methods, and be reported to the National Infectious Disease Reporting Information System (NIDRIS), which will be reviewed by the disease control department.

Under the background of COVID-19, people who entered China were subject to a series of controls during the coronavirus outbreak. Those who had been infected with SARS-CoV-2 or presented symptoms of COVID-19 were declared to the authority and were transferred to the designated medical institution on the day of entry for further investigation. All the entry personnel were subject to “intensive quarantine for 14 days + home quarantine for 7 days + self-health monitoring for 7 days,” and the quarantine policy was adjusted regarding different situations. The health status of the inbound personnel was monitored daily, with the nose, pharynx, and pharyngeal sputum swab sampling at 3, 7, and 14 days (some also need on the 21st day of entry) after entering for detection of SARS-CoV-2. Once found relevant symptoms or detected SARS-CoV-2, the patient was immediately closed-loop transported to a designated hospital for treatment.

### Case Confirmation and Management

Since China has been certified as a malaria-free country on 30 June 2021, early reporting and diagnosis confirmation of imported malaria cases are of great importance to consolidate the hard-won achievement. For presumed malaria cases, when any kind of laboratory confirmation was applied (RDT or microscopy method), the medical institution reported the case to the NIDRIS. The laboratory confirmation results were reviewed by a peer or superior Center of Disease Control and Prevention (CDCs) and finally confirmed by CDCs at the provincial level. Both microscopy and PCR methods were used to confirm the diagnosis and *plasmodium* typing. For this case, the provincial CDC used direct microscopic examination and a multiplex quantitative real-time PCR (qPCR) for malaria confirmation.

The diagnosis of COVID-19 followed a national unified standard. One is defined as a person with suspected infection if had a history of contacting with patients with COVID-19 or traveling history to communities where COVID-19 has been reported if the patient has fever, respiratory symptoms, or other laboratory test results supporting infection. A confirmed COVID-19 case is a suspected patient with a positive result of a nucleic acid of SARS-CoV-2 or isolation of SARS-CoV-2. Techniques for SARS-CoV-2 confirmation include real-time fluorescent PCR (RT-PCR) and virus genome sequencing. To resolve the limitations of qRT-PCR testing and difficult COVID-19 suspected cases, serological testing (immunoglobulin M/immunoglobulin G [IgM/IgG] antibody detection) is suggested as a complementary identification assay (5, 6).

### Epidemiological Investigation

Epidemiological investigation of malaria is finished in 3 days, after the case being reported to the NIDRIS. The following needs to be investigated with the patient: age, gender, address, the progress of disease onset (clinical manifestations, complications, date of onset, fever, severity of disease, location of onset, laboratory test results, etc.), diagnosis (laboratory test result, time, and institution of diagnosis), current treatment, possible sources of infection (overseas residence history, use of preventive measures, blood transfusion history, whether family members or visiting relatives and friends have malaria-related symptoms, etc.), and case follow-up.

Except for the demographic information mentioned above, content of the epidemiological investigation of COVID-19 includes disease onset and medical treatment, epidemiological history (investigation of infection source, close contact, and exposure site), and laboratory test results.

### Ethics Approval and Consent to Participate

The studies involving human participants were reviewed and approved by the Ethics Committee of Hangzhou Xixi Hospital (2021 No. 15).

## Results

### Case Presentation

**Figure 1:**
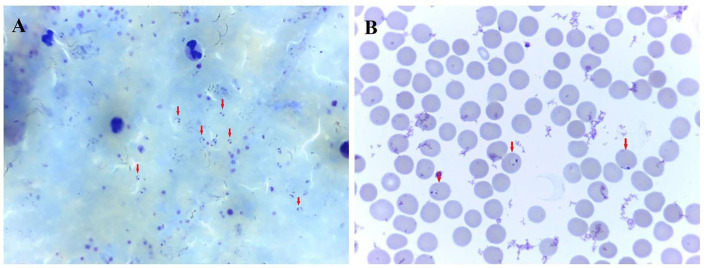
Photomicrograph showing trophozoite of *P. falciparum* malaria. **(A)** thick blood smear. **(B)** thin blood smear. A certain number of Plasmodium parasites can be seen on the thick blood smear. The classic, ring-shaped trophozoites of *P. falciparum* parasite were shown in a thin blood smear.

The patient reported fever with chills, about 20 h after landing in China and was transported by special bus to a hotel in uptown for isolation. Body temperature of the patient was up to 41°C. The patient also reported fatigue, headache, bilateral lower extremity muscle pain, stomach cramp, and slightly nausea without vomiting on the same day. The patient was immediately sent to the local infectious disease hospital by a special ride for further examination. A physical examination revealed a body temperature of 38.2°C, blood pressure of 106/65 mm Hg, the pulse of 99 beats per minute, respiratory rate of 21 breaths per minute, and oxygen saturation of 97% while breathing ambient air. Laboratory tests revealed decreased lymphocyte counts, increased C-reactive protein concentrations, increased serum amyloid A concentrations, and slightly increased D-dimer, fibrinogen, and glucose levels (Table 1). A blood smear examination revealed a positive result for *P. falciparum* parasitemia on 18 June, which was confirmed by Zhejiang Provincial Center for Disease Control and Prevention (Figure 1).

**Table 1:** Initial laboratory results for the first hospitalization.

**Variable**	**Reference range**	**Result**
Red–cell count (10^9^/L)	3.80–5.10	4.69
Hemoglobin (g/L)	115–150	148
White–cell count (10^9^/L)	3.50–9.50	3.36↓
Platelet count (10^9^/L)	125–350	137
Absolute lymphocyte count (10^9^/L)	1.10–3.20	0.31↓
Absolute Eosnophils count (10^9^/L)	0.02–0.52	0.01↓
Neutrophil ratio (%)	40.0–75.0	82.8↑
SAA (mg/L)	0–10	38.44↑
C–reactive protein (mg/L)	0–10	17.35↑
Procalcitonin ng/mL	0–0.5	0.652↑
D–dimer (μg/mL)	0.00–0.55	1.98↑
CPK (U/L)	24–170	42
Glucose (mg/dL)	3.90–6.10	6.74
Creatinine (mg/dL)	41–81	51
Blood urea (mmol/L)	2.6–7.5	3.8
Total bilirubin (μmol/L)	3–22	16
Aspartate aminotransferase (U/L)	14–36	39↑
Alanine aminotransferase (U/L)	9–52	19
Sodium (mmol/L)	136–146	139
Potassium (mmol/L)	3.4–5.5	3.7
Calcium (mmol/L)	1.15–1.29	1.14↓
Chloride (mEq/L)	98–106	105

*The upward arrow ("↑") indicates the result was above the reference range of the specific variable. On the contrary, the downward arrow ("↓") indicates the result of the variable was below the reference range*.

The patient was administered with dihydroartemisinin piperaquine tablets (2 tablets at 0 h−6 h−24 h−32 h), according to the technical regulation for the application of antimalarials in China, following the principles of safety, effectiveness, rationality, and standardization. On 19 June, the conventional SARS-CoV-2 PCR test for travelers who have been abroad revealed that the patient was SARS-CoV-2 positive, using the nasopharyngeal swab sample. The patient was administered with Lopinavir and Ritonavir tablets (400 mg bis in die, b.i.d.), Arbidol (0.2 g ter in die, t.i.d.), recombinant human interferon α-2b (500 × 105 U b.i.d.), and Compound Maxing Yifei mixture (a traditional Chinese patent medicine).

**Figure 2:**
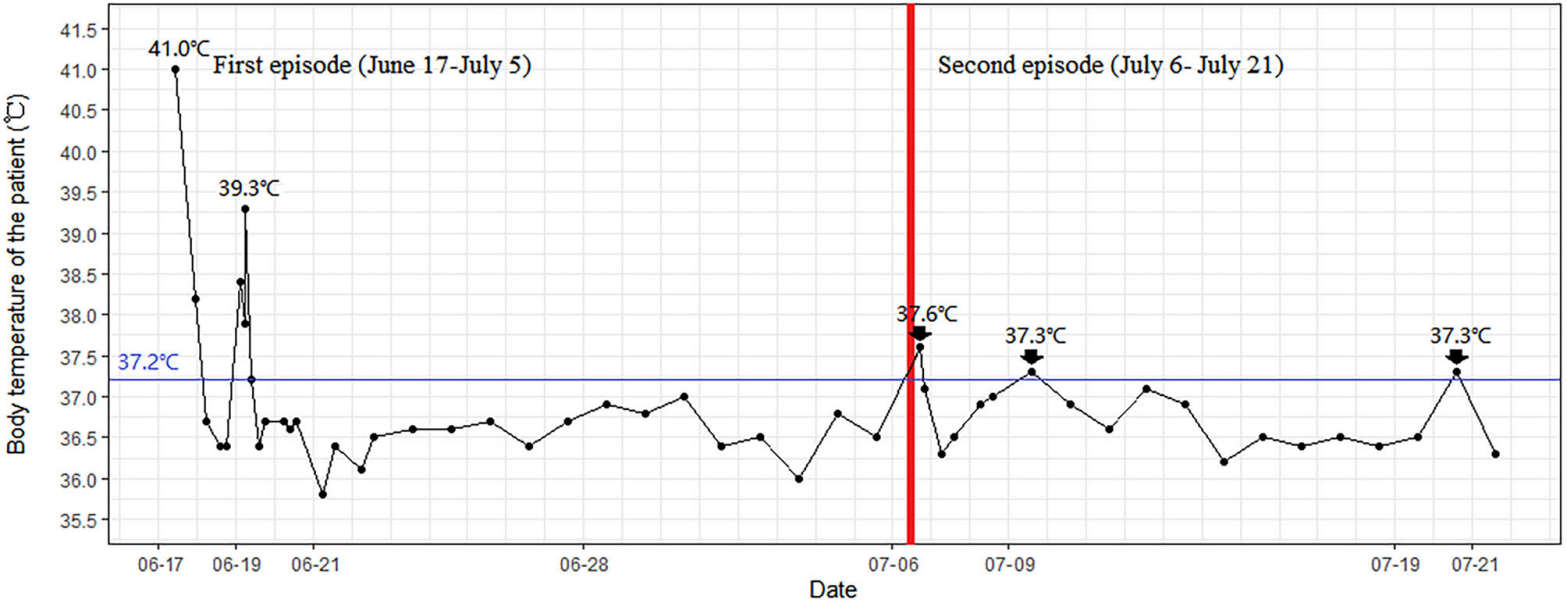
The temperature curve of the patient.

On 20 June, the body temperature of the patient was dropped to 36.3°C and maintained at the normal level (Figure 2). On 22 June, the blood smears examination for malaria was negative and remained negative on 25 June and 5 July. The SARS-CoV-2 PCR results during her hospital stay were negative multiple times. On 4 and 5 July, the nasopharyngeal SARS-CoV-2 PCR tests remained negative, with an interval between the two tests of >24 h. Thus, the patient met China's COVID-19 discharge standards and left the hospital on 5 July.

**Figure 3:**
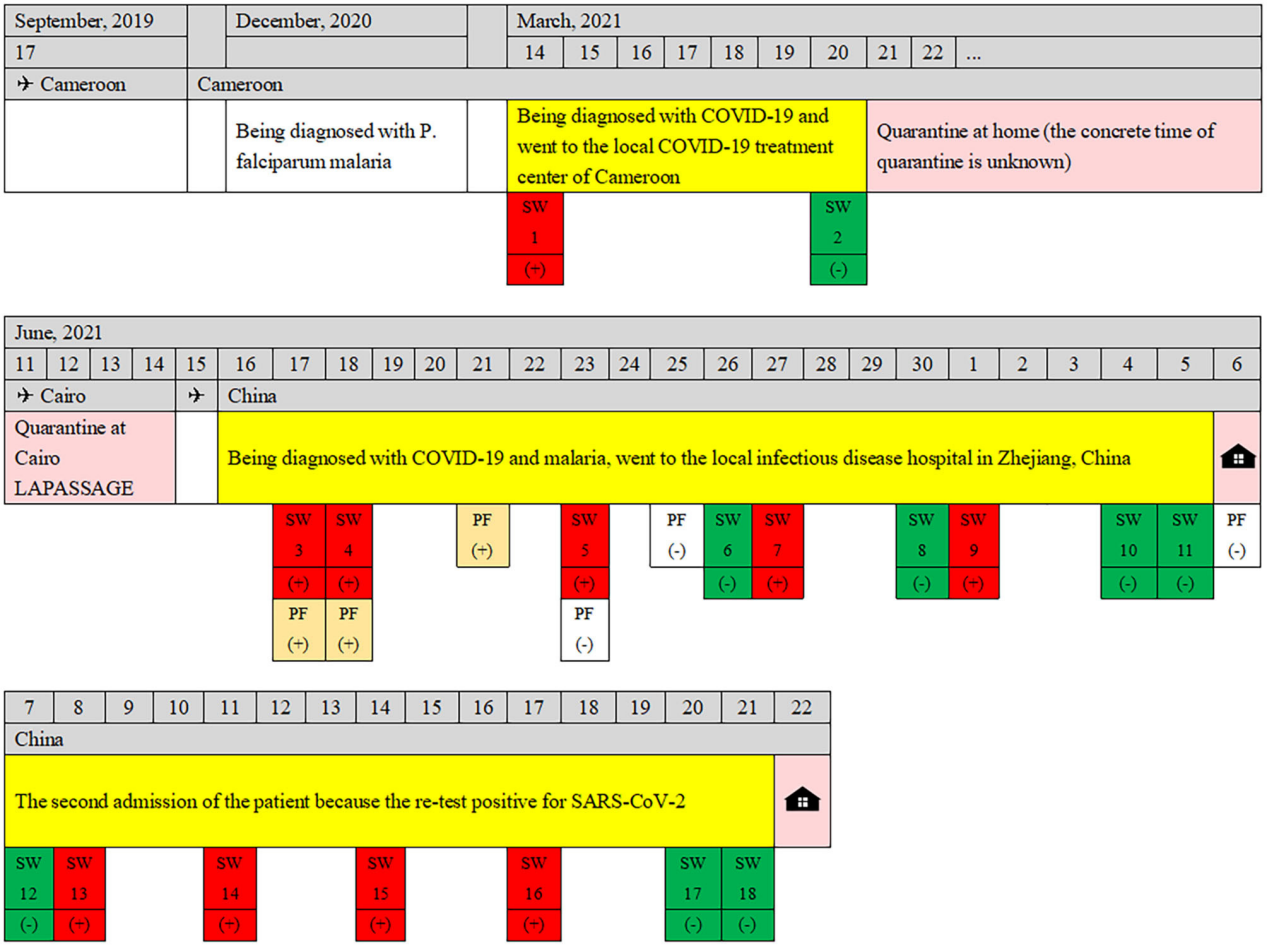
The diagram of the complete course of disease for the patient.

On 6 July, however, the patient had a low-grade fever (37.6°C) again, with anxiety and insomnia. She was soon been hospitalized, with laboratory tests indicating no evidence of inflammatory reactions (Table 2). This time, she was treated with Zolpidem Tartrate tablets, Diazepam, Paroxetine Hydrochloride tablets, Thymosin α1, and Lianhua Qinwen Jiaonang (another traditional Chinese patent medicine). Using nasopharyngeal swabs with dual reagents, the patient tested positive for COVID-19 on 8, 11, 14, and 17 July and negative on 20 and 21 July. As the patient met China's COVID-19 discharge standards, she was discharged from the hospital. The patient's vital signs were stable and her mental state improved before discharge. The complete course of disease for the patient is shown in Figure 3.

**Table 2:** Initial laboratory results for the second hospitalization.

**Variable**	**Reference range**	**Result**
Red–cell count (10^9^/L)	3.80–5.10	4.12
Hemoglobin (g/L)	115–150	127
White–cell count (10^9^/L)	3.50–9.50	4.52
Platelet count (10^9^/L)	125–350	276
Absolute lymphocyte count (10^9^/L)	1.10–3.20	1.55
Absolute Eosnophils count (10^9^/L)	0.02–0.52	0.08
Neutrophil ratio (%)	40.0–75.0	54.0
SAA (mg/L)	0–10	15.18↑
C–reactive protein (mg/L)	0–10	9.98
Procalcitonin ng/mL	0–0.5	0.23
D–dimer (μg/mL)	0.00–0.55	0.33
CPK (U/L)	24–170	32
Glucose (mg/dL)	3.90–6.10	8.80↑
Creatinine (mg/dL)	41–81	41
Blood urea (mmol/L)	2.6–7.5	3.6
Total bilirubin (μmol/L)	3–22	13
Aspartate aminotransferase (U/L)	14–36	33
Alanine aminotransferase (U/L)	9–52	26
Sodium (mmol/L)	136–146	138
Potassium (mmol/L)	3.4–5.5	3.7
Calcium (mmol/L)	1.15–1.29	1.18
Chloride (mEq/L)	98–106	106↑

*The upward arrow ("↑") indicates the result was above the reference range of the specific variable. On the contrary, the downward arrow ("↓") indicates the result of the variable was below the reference range*.

### Epidemiological Background

According to the epidemiological investigation, the patient has been working in Cameroon since February 2014, travel to China occasionally to visit friends and relatives, and has never been back to China since September 2019. She was diagnosed with *P. falciparum* malaria in December 2020 and was treated with an unspecified therapeutic regimen. On 14 March 2021, the patient developed fatigue and dysgeusia, and her nasopharyngeal SARS-CoV-2 PCR test was positive. She attended a local COVID-19 treatment center, was treated with an unspecified COVID-19 drug regimen, and was discharged from the COVID-19 treatment center on 20 March 2021, and underwent home isolation as her nasopharyngeal SARS-CoV-2 PCR was negative. SARS-CoV-2 PCR tests were negative on re-examination on 29 March, 17 May, 8 June, and 9 June. On 11 June, she flew on flight MS888 from Cameroon to Cairo. After being quarantined at a hotel in Cairo for 4 days, she took flight MS953 and arrived in China on June 15. She arrived at Hangzhou Xiao-Shan International Airport at 14:40 on 16 June. On 17 June, the day after she landed in China and was quarantined, she reported fever (41°C) with chills, fatigue, headaches, muscle aches, stomach cramps, and slight nausea and was immediately sent to the local infectious disease hospital for a special ride for further examination.

## Discussion

This is the first case of SARS-CoV-2 and *P. falciparum* co-infection reported in China. Our report adds to the growing body of literature on co-infections between malaria and COVID-19, which have also been reported in Dubai (7), India (8, 9), Indonesia (10, 11), Qatar (12), Turkey (13), and Portugal (14). Common symptoms of co-infection with SARS-CoV-2 and malaria included high fever with chills, profuse sweating, running nose, sore throat, cough, short breath or breathing difficulty, abdominal pain, nausea, vomiting, headache, loss of smell/taste, etc. These symptoms are very common in many other infectious diseases. Symptoms of COVID-19 and malaria are overlapping to a large extent (15, 16). Complications, such as acute respiratory distress syndrome (ARDS), septic shock, and multi-organ failure, can also occur from both malaria and COVID-19 (17), thus these reports all emphasize the possibility of misdiagnoses between malaria and COVID-19. Most patients have a good prognosis after treatment, however, a previously healthy 28-year-old woman co-infected with SARS-CoV-2 and *P. falciparum* in India developed severe disease indications in a short time period, showed neurological symptoms, and passed away 6 days after the disease onset (18). This case showed the potential danger of co-infection, especially in a low resource setting.

The delayed diagnosis may lead to an increase in disease severity and cause catastrophic events. Early diagnosis of malaria and other infectious diseases is of great importance in the context of the COVID-19 pandemic. There might be three types of mistakes in diagnosis and treatment: (1) a negative result for COVID-19 while overlooking the malarial infection; (2) being tested for malaria when a patient has a COVID-19 infection; and (3) being infected with both pathogens, but being misdiagnosed for one. A misdiagnosis of both the diseases could result in the spread of the disease and a poor prognosis for the patient. Since COVID-19 is of high priority currently, pre-entry quarantine and RT-PCR for SARS-CoV-2 have been a routine in many countries. If the RT-PCR reported a positive result, probable malaria (or other infectious diseases) is easily overlooked and might lead to a poor prognosis. In China, a series of regulations for imported malaria prevention and control was made, i.e., the cooperation of medical institutions and CDCs for case diagnosis and management, and the “1-3-7” approach for malaria case reporting, confirmation, investigation, and appropriate public health response (5). For those who were suspected of malaria, after they showed symptoms (such as fever) and sought medical service, medical institutions must identify cases using RDT and microscopy methods. All the positive samples must be verified at the county, prefectural, and provincial laboratories. Our patient showed symptoms 20 h after landing in China. Considering the incubation period, she was most likely infected with *P. falciparum* before she came into China and was defined as an imported malaria case. This case once again highlights that asking about epidemiological history is as important as maintaining competency in malaria diagnosis.

Another important clue is the fever pattern. Malaria is characterized by recurring paroxysms of what are known as ague, in which, as a rule, chill, fever, and sweat follow each other in an orderly sequence (19). Fever may rise to 40 or 41°C with chills, nausea, and vomiting being common. For patients with immunodeficiency (20) or self-medication with anti-malarial drugs, fever may be atypical or absent. As for COVID-19, with the exception of the 1.6–56.0% of asymptomatic patients (21), 79.43% developed fever, among whom 38.16% were low grade and 44.33% were medium grade (22). The relatively low proportion of COVID-19 patients with high-grade fever was different from that observed in patients with malaria. Another difference is that unlike malaria, the COVID-19 fever does not occur with the regularity. Therefore, fever patterns and the patients' immune status can be considered the markers to differentiate the diagnosis of COVID-19 from malaria.

This patient has been diagnosed with both *P. falciparum* malaria and COVID-19 in December 2020 and March 2021, respectively. Because of the lack of information on SARS-CoV-2 strains of the March and June episodes, we could not conclude that it was a re-infection for SARS-CoV-2 or relapse. Therefore, as malaria, because malaria symptoms appeared only 6 days after she left Cameroon and the time was close to the minimum incubation period. However, there is a possibility that prior treatments failed to remove all parasites from the patient's body, and recurrence occurred when the patient experienced reduced immunity due to the long journey and the substantial pressure from the COVID-19 pandemic. If true, the immune response of the patient might play an important role in the emergence, development, recrudescence and re-test positive of COVID-19 and malaria (23, 24). Another possible explanation is that the COVID-19 might induce the recrudescence for *P. falciparum* malaria, since literature about the possible relationship between COVID-19 infection and relapse of *Plasmodium vivax* (*P. vivax*) and *Plasmodium ovale (P. ovale*) malaria was reported. Although the interaction between malaria and COVID-19 remains unknown yet, some potential biological link between *P. falciparum* infection and SARS-CoV-2 infection or severity has been reported (25), such as the share of ACE2 and CD147 as an entry point of host cells. In addition, other literature has indicated that the delayed presentation of malaria could be associated with stress, pregnancy, and immunosuppression (26), and so as COVID-19. When it comes to severe cases, it is known that the same mechanism of ARDS in malaria and COVID-19 could lead to the rapid deterioration and poor prognosis upon co-infection (17). The association between COVID-19 and malaria is complicated and further studies were needed.

Reasons that recovered patients with COVID-19 test positive for SARS-CoV-2 are complex that include re-infections, false-negative test results when discharged from the hospital, not completely meeting the discharge criteria or incomplete elimination of the virus (27, 28). The patient in this case had a history of being diagnosed with *P. falciparum* malaria, and due to the strict anti-epidemic measures in China, samples are obtained from multiple sites from each suspected patient with COVID-19 and RT-PCR tests are performed multiple times. In our clinic, we found that a higher rate of patients were redetectable as positive (RP) in the second year of the COVID-19 pandemic when compared with the first year. Although most COVID-19 RP patients were not infectious, it remains important to perform regular SARS-CoV-2 RNA testing and follow-up assessments of infectivity. Another non-neglectable effect is the emotional consequence due to the COVID-19 pandemic. According to a recent review, numerous emotional outcomes, such as stress, depression, irritability, insomnia, fear, confusion, anger, frustration, boredom, and stigma, are associated with quarantine, some of which persisted after the quarantine was lifted. We emphasize the relationship between patients' emotional status and disease progression, especially how they interact with each other, because of the potential health consequences this case showed.

With the impact of the COVID-19 pandemic, disruptions to healthcare systems and malaria control programs may lead to a higher incidence of malaria infections (29), and thus, co-infections with SARS-CoV-2 may become more common as well. Observational studies are needed to evaluate this potential problem to discuss the epidemiology, mechanism, clinical treatment, and outcome of SARS-CoV-2 and malaria co-infection. The COVID-19 pandemic could interrupt malaria prevention campaigns, eliminating the progress made over the past 5 years. The WHO, the United Nations International Children's Emergency Fund (UNICEF) and the International Federation of Red Cross (IFRC) have published an interim guidance document to advise on how to sustain essential services for patients with malaria at community levels while ensuring that the response to COVID-19 is effective. The report also provides practical recommendations to help keep communities and health workers safe, to sustain essential services at the community level, and to ensure an effective response to COVID-19 (2).

Our study has several limitations. Since it is a case report, it may not be generalizable because only one case is not enough to represent all the cases. Second, viruses, such as SARS-CoV-2, continuously evolve as changes in the genetic code occur during the replication of the genome. New SARS-CoV-2 variants of concern (VoC) could have quite different characteristics in the aspects of virulence, transmissibility, and ability to escape naturally acquired and vaccine-induced immunity. Further studies were needed when more co-infected cases were collected, to observe the symptoms, monitor the possible undesirable outcomes, and seek appropriate diagnosis and treatment strategies.

To conclude, the patient was finally diagnosed with co-infection of both SARS-CoV-2 and *P. falciparum*, mostly infected abroad since she was quarantined as soon as she landed in China. This case adds to our knowledge of the presentation and treatment of SARS-CoV-2 co-infections with malaria. We suggest that clinicians consider the epidemiological histories of patients, especially those who have a history of traveling to malaria-endemic areas, such as Africa and Southeast Asia. Clinicians should also conduct malaria screening to detect possible cases as soon as possible and avoid possible missed diagnoses of COVID-19 co-infections. High fever is an important feature for distinguishing malaria from COVID-19 and further studies are required to reveal whether COVID-19 and malaria co-infections lead to greater severity and worse outcomes.

## Data Availability Statement

**T**he original contributions presented in the study are included in the article/supplementary material, further inquiries can be directed to the corresponding author/s.

## Ethics Statement

Written informed consent was obtained from the individual(s) for the publication of any potentially identifiable images or data included in this article.

## Author Contributions

QH, K-NP, L-NY, and WR contributed to the conception of the study. QH and K-NP performed the diagnosis and treatment of the patient. H-LC and J-QZ contributed significantly to the Plasmodium detection, reexaming, and typing. W-JX, X-XW, and XZ performed the analyses and wrote the manuscript. L-NY and WR helped perform the analysis with constructive discussions. All authors contributed to the article and approved the submitted version.

## Funding

This work was supported by the Zhejiang Provincial Medical and Health Project (2020PY038). The funders had no role in the design of the study and collection, analysis, and interpretation of data and in writing the manuscript.

## Conflict of Interest

The authors declare that the research was conducted in the absence of any commercial or financial relationships that could be construed as a potential conflict of interest.

## Publisher's Note

All claims expressed in this article are solely those of the authors and do not necessarily represent those of their affiliated organizations, or those of the publisher, the editors and the reviewers. Any product that may be evaluated in this article, or claim that may be made by its manufacturer, is not guaranteed or endorsed by the publisher.
